# The Relationship Among Probable SARCopenia, Osteoporosis and SuprasPinatus Tendon Tears in Postmenopausal Women: The SARCOSP Study

**DOI:** 10.1007/s00223-024-01183-7

**Published:** 2024-02-12

**Authors:** Murat Kara, Özgür Kara, Mahmut Esad Durmuş, Pelin Analay, Fatıma Edibe Şener, Beyza Nur Çıtır, Gizem Olgu Korkmaz, Zeliha Ünlü, Tülay Tiftik, Eda Gürçay, Cevriye Mülkoğlu, Berkay Yalçınkaya, Fatih Bağcıer, Mahmud Fazıl Aksakal, Kübra Erdoğan, Ahmet Sertçelik, Banu Çakır, Bayram Kaymak, Levent Özçakar

**Affiliations:** 1 Department of Physical and Rehabilitation Medicine, Hacettepe University Medical School, Ankara, Turkey; 2 Department of Physical Medicine and Rehabilitation, Abdurrahman Yurtaslan Oncology Training and Research Hospital, Ankara, Turkey; 3 Department of Physical Medicine and Rehabilitation, Celal Bayar University, Manisa, Turkey; 4 Department of Physical Medicine and Rehabilitation, Ankara Training and Research Hospital, Ankara, Turkey; 5 Department of Physical Medicine and Rehabilitation, University of Health Sciences, Gaziler Physical Medicine and Rehabilitation Education and Research Hospital, Ankara, Turkey; 6 Department of Physical Medicine and Rehabilitation, Basaksehir Cam Sakura Hospital, Istanbul, Turkey; 7 Department of Public Health, Division of Epidemiology, Hacettepe University Medical School, Ankara, Turkey

**Keywords:** Rotator cuff, Grip strength, Ultrasound, Hypertension, Bone

## Abstract

We aimed to investigate the relationship among probable sarcopenia, osteoporosis (OP) and supraspinatus tendon (SSP) tears in postmenopausal women. Postmenopausal women screened/followed for OP were recruited. Demographic data, comorbidities, exercise/smoking status, and handgrip strength values were recorded. Probable sarcopenia was diagnosed as handgrip strength values < 20 kg. Achilles and SSP thicknesses were measured using ultrasound. Among 1443 postmenopausal women, 268 (18.6%) subjects had SSP tears. Unilateral tears were on the dominant side in 146 (10.1%) and on the non-dominant side in 55 women (3.8%). In contrast to those without, women with SSP tears had older age, lower level of education, thinner SSP and lower grip strength (all p < 0.05). In addition, they had higher frequencies of hypertension, hyperlipidemia, DM, OP and probable sarcopenia, but lower exercise frequency (all p < 0.05). Binary logistic regression modeling revealed that age [odds ratio (OR): 1.046 (1.024–1.067 95% CI)], hypertension [OR: 1.560 (1.145–2.124 95% CI)], OP [OR: 1.371 (1.022–1.839 95% CI)] and probable sarcopenia [OR: 1.386 (1.031–1.861 95% CI)] were significant predictors for SSP tears (all p < 0.05). This study showed that age, presence of hypertension, probable sarcopenia and OP were related with SSP tears in postmenopausal women. To this end, although OP appeared to be related to SSP tears, SSP tear/thickness evaluation can be recommended for OP patients, especially those who have other risk factors such as older age, higher BMI, hypertension, and probable sarcopenia.

## Introduction

Rotator cuff (RC) tears are common causes of shoulder pain and upper limb dysfunction—affecting daily life activities and causing disability. The prevalence of partial-thickness RC tear is 13–37% and increases with age [1]. Supraspinatus tendon (SSP) tears are the most common RC injuries, with a prevalence of 22.2% (for full-thickness tears) in a cohort of women aged between 64 and 87 years [2]. Of note, partial-thickness tears and disuse atrophy of the musculoskeletal structures around the shoulder joint may affect tendon healing adversely—leading to full-thickness tears and further complicating treatment [3]. Although the pathogenesis of non-traumatic SSP tears remains unclear; age, smoking, diabetes mellitus (DM), hyperlipidemia, hypertension and genetic factors were reported to be in association with SSP tears [4].

On the other hand, since estrogen receptors are expressed in bones, tendons, muscles and ligaments; its deficiency in the postmenopausal period can have negative impact on those structures [5, 6] Likewise, RC tear would be a relevant example in the elderly postmenopausal women [7]. Several studies have reported a relationship between RC tears, sarcopenia [8] and osteoporosis (OP) in the humeral greater tuberosity [9–11]. A recent large-scale longitudinal study revealed that the risk of RC tears was about 1.8-fold higher in OP patients than those without OP [12]. Moreover, failure of tendon repair has also been reported as high as 68% whereby bone quality/quantity of the humeral greater tuberosity is thought to be one of the factors affecting repair integrity [13].

Furthermore, it has been reported that sarcopenia is related with increased risks of rotator cuff tendon diseases in older adults [8]. Others include age, tear size, fatty infiltration, muscle atrophy/retraction, smoking and DM [8, 14]. Indeed, it has been shown that sarcopenia is related to the biochemical dysregulation of the renin-angiotensin system (RAS). The latter can be considered a complex biochemical pathway largely involved in the protein turnover, cellular apoptosis, and anabolic processes of collagen fibers (i.e., the collagen metabolism) of different anatomical structures such as the tendons of the musculoskeletal system. Moreover, dysregulation of both the classical and non-classical RAS axes may lead to hypoperfusion of the muscle–tendon units promoting degenerative changes of the tendon tissue (with reduced resistance to load) and increasing the risk of disruption [15].

To our best knowledge; there is no comprehensive study evaluating the relationship of SSP tears with OP, sarcopenia and the aforementioned clinical factors together in the literature. In addition, as OP and sarcopenia are more commonly reported in women [16], the aim of this study was to explore the association among (probable) sarcopenia, OP, and clinical variables with SSP tendon tears in postmenopausal women.

## Methods

### Subjects and Design

This cross-sectional study was performed in six tertiary-care hospitals. Postmenopausal women screened/followed for OP were recruited. Those who had any organ failure, neuromuscular and rheumatic diseases, history of major orthopedic surgery (i.e., total knee or hip joint replacement) or secondary OP were excluded. Informed consent obtained from all individual participants included in the study. The study was conducted according to the guidelines of the Declaration of Helsinki and was approved by the local Ethics Committee of Hacettepe University Medical School. Demographic data including age, body mass index (BMI), education level (in years), exercise and current smoking statuses, and accompanying comorbidities (e.g. hypertension, DM, hyperlipidemia) were recorded. Subjects were considered to be doing exercise if they performed moderate-intensity of physical activity or walking (> 30 min/day) at least 3–4 days/week [17].

### Osteoporosis Evaluation

The measurements were performed from anteroposterior lumbar vertebrae (L1-4), femoral total and femoral neck regions using dual energy X-ray absorptiometry (DXA) scanners (GE Lunar scanner; GE Healthcare, Madison, WI, USA, Hologic Explorer; Hologic Inc. scanner, Bedford, USA, Primus; OsteoSys, Seoul, S. Korea, and FDX Visionary; Fujifilm, Tokyo, Japan). The diagnosis of OP was defined as a T-score of ≤ −2.5 SD for BMD at lumbar vertebrae and/or femoral total or femoral neck [18]. While calculating the mean L1-L4 BMD T-scores, abnormal scores (i.e. more than 1.0 SD difference between T-scores of consecutive vertebrae) were excluded, and the mean T-score value of the other vertebrae (three or at least two) was considered for the diagnosis of OP [19].

### Ultrasonographic Measurements

**Fig. 1:**
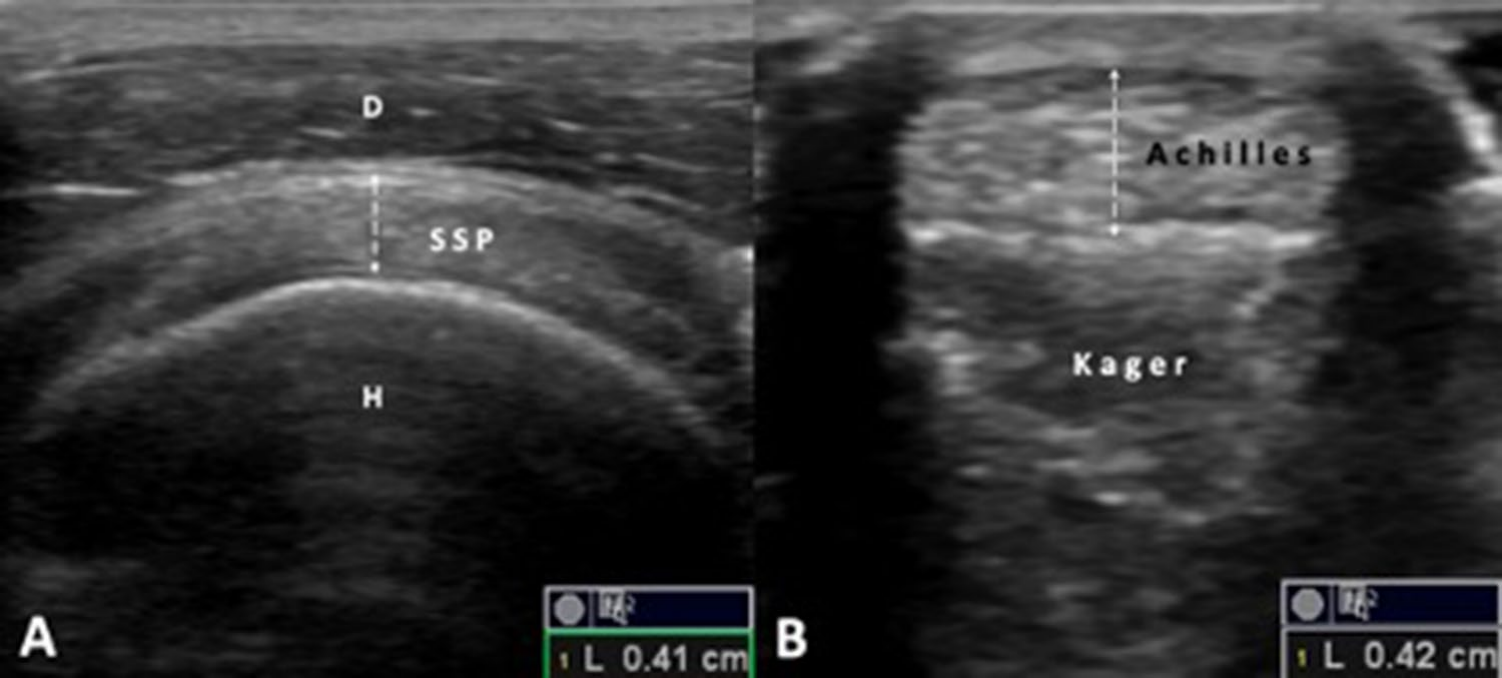
Ultrasonographic measurements of the supraspinatus (SSP). **A** and Achilles **B** tendons during axial imaging. *H* humerus, *D* deltoid muscle, Kager: fat pad

Ultrasound (US) measurements were acquired using 5–12 MHz (Logiq P5, GE, Medical Systems, USA), 6–11 MHz (Nemio XH, Toshiba, Japan), 8–10 MHz (Mindray DC-7, Mindray Bio-medical Electronics, Shenzhen, China), 5–12 MHz (Logiq E, GE Healthcare, China), 6–11 MHz (Nemio XH, Toshiba, Japan), 4–13 MHz Clarius L7 (Clarius Mobile Health, 130–2985 Virtual Way, Vanvouer, BC, Canada) linear probes from the dominant hand sides of the subjects. SSP thickness measurements were performed in the Crass position i.e. sitting face-to-face with the patients as the dorsum of their hands were kept over the ipsilateral hip and their shoulders were in hyperextension and internal rotation [20]. Tendon thickness was measured axially at its thickest point—as the distance between the humeral articular (hyaline) cartilage and the subdeltoid bursa (Fig. 1A). In case of partial- or full-thickness SSP tears, SSP tendinopathy or subdeltoid bursitis, the measurement was taken from the non-dominant hand side. In case of bilateral SSP tendinopathy/tears, the measurements were not acquired from the SSP. During Achilles tendon measurements, the participants were lying in prone position while their feet were hanging out of the examination table. Tendon thickness was measured axially (thickest point between the epitenons) at the level of lateral malleolus (Fig. 1B) [21]. All physicians who performed the measurements had at least 2–3 years of experience in musculoskeletal US and all images were reviewed (by the senior authors) for technical/quality standardization before pertinent data were enrolled in the study.

### Functional Evaluation

As the diagnosis of sarcopenia includes low muscle mass and function, detection of low muscle function (i.e. grip strength and/or chair stand test) leads to a diagnosis of “probable” sarcopenia. Once low muscle function is detected, adjusted sonographic thigh muscle measurement is used for early diagnosis/confirmation of sarcopenia. Presence of a mobility limitation, i.e. gait speed ≤ 0.8 m/s and/or inability to rise from a chair without support leads to the diagnosis of “severe” sarcopenia [22]. Accordingly, grip strength measurement was performed as an indicator of low muscle function. Handgrip strength measurement was performed from the dominant hand side using a Jamar hydraulic hand dynamometer (Baseline Hydraulic Hand dynamometer Irvington, NY, USA). Three measurements were taken whereby the maximum values were used for the analyses. The diagnosis of “probable sarcopenia” was accepted as handgrip strength values < 20 kg [23].

### Statistical Analysis

Frequency/percent distributions (%), and mean ± SD are presented for categorical and continuous variables, as appropriate. Normality assumption was tested by Kolmogorov–Smirnov test. Group comparisons were based on Student t or Mann–Whitney U tests, as appropriate. Categorical variables were compared by Chi-square test. Possible associations between SSP tears and significant clinical parameters (i.e., age, BMI, education level, smoking, doing exercise, hypertension, hyperlipidemia, DM, probable sarcopenia and OP) were investigated with binary logistic regression analyses by using enter methods. Hosmer–Lemeshow goodness‐of‐fit statistics was used to evaluate models fit. Statistical significance was set at p < 0.05*.* All analyses were conducted using SPSS statistical software, version 23.0.

## Results

A total of 1443 postmenopausal women (aged between 45 and 87 years) were consecutively enrolled. 268 women (18.6%) had SSP tears and 67 of them (4.6%) were bilateral. Unilateral tears were on the dominant side in 146 (10.1%) and on the non-dominant side in 55 women (3.8%). Comparison of the clinical findings between women with and without SSP tears are shown in Table 1. In contrast to those without, women with SSP tears had older age, higher BMI, lower level of education, thinner SSP and lower grip strength (all p < 0.05). In addition, they had higher frequencies of hypertension, hyperlipidemia, DM, OP and probable sarcopenia, but lower exercise frequency (all p < 0.05). Smoking status and Achilles tendon thicknesses were found to be similar between the groups (both p > 0.05). While 547 (37.9%) of 1443 women were diagnosed with OP, 156 of them (28.5%) were under antiresorptive treatment, and 391 (71.5%) were newly diagnosed.

**Table 1:** Clinical characteristics of the subjects (N = 1443)

	With SSP tear (N = 268)	Without SSP tear (N = 1175)	P
Age (yr)	64.2 ± 7.7	60.4 ± 7.2	** < 0.001**
BMI (kg/m^2^)	31.4 ± 5.7	30.1 ± 5.4	**0.004**
Exercise	40 (14.9)	280 (23.8)	**0.002**
Education (yr)	5.7 ± 4.3	7.0 ± 4.6	** < 0.001**
Current smoking	40 (14.9)	210 (17.9)	0.250
*Comorbidities*			
Hypertension	174 (64.9)	530 (45.1)	** < 0.001**
Hyperlipidemia	67 (25.0)	203 (17.3)	**0.003**
DM	100 (37.3)	349 (29.7)	**0.015**
Osteoporosis	124 (46.3)	423 (36.0)	**0.002**
Grip strength (kg)	20.4 ± 4.9	22.1 ± 4.8	** < 0.001**
Probable sarcopenia*	113 (42.2)	328 (27.9)	** < 0.001**
SSP tendon (mm)	4.8 ± 1.0	5.1 ± 1.0	** < 0.001**
Achilles tendon (mm)	4.5 ± 0.7	4.4 ± 0.8	0.130

Data are presented as mean ± standard deviation, N (%)

*SSP* supraspinatus tendon, *yr* year, *BMI* body mass index, *DM* diabetes mellitus, *OP* osteoporosis, *BMD* bone mineral densitometry,

Statistically significant variables are shown as bold

^*^ Probable sarcopenia was accepted as grip strength < 20 kg

Binary logistic regression modeling (Table 2) revealed that age [odds ratio (OR): 1.046 (1.024–1.067 95% CI)], hypertension [OR: 1.560 (1.145–2.124 95% CI)], OP [OR: 1.371 (1.022–1.839 95% CI)] and probable sarcopenia [OR: 1.386 (1.031–1.861 95% CI)] were significant predictors for SSP tears (all p < 0.05). When the absolute values of grip strength were entered into the binary regression analysis, grip strength was found to be negatively (0.967 (0.937–0.997) associated with SSP tear (p = 0.034). When OP was entered into the regression analysis as newly diagnosed and under treatment; compared to those without OP, patients under treatment were positively associated with SSP tear [OR: 1.705 (1.092–2.663)](p = 0.019]. However, this relationship was not detected in those who were newly diagnosed [OR: 1.314 (0.957–1.806)] (p = 0.092).

**Table 2:** Binary logistic regression analyses between clinical variables and SSP tear (N = 1443)

Variables	OR	95% CI	P
Age	1.046	1.024–1.067	** < 0.001**
BMI	1.026	0.999–1.055	0.063
Education	0.922	0.809–1.051	0.225
Exercise	0.730	0.498–1.070	0.107
Smoking	1.113	0.754–1.645	0.590
Hypertension	1.560	1.145–2.124	**0.005**
DM	0.964	0.695–1.336	0.824
Hyperlipidemia	1.166	0.814–1.669	0.402
Osteoporosis	1.371	1.022–1.839	**0.031**
Probable sarcopenia*	1.386	1.031–1.861	**0.025**

*SSP* supraspinatus tendon, *OR* odds ratio, *CI* confidence interval, *BMI* body mass index, *DM* diabetes mellitus

Statistically significant variables are shown as bold

^*^Probable sarcopenia was accepted as grip strength < 20 kg

## Discussion

To the best knowledge of the authors, this is the first study to report on concrete association between SSP tendon tears and clinical conditions including OP and probable sarcopenia. Notably, the incidence of SSP tears increased with age. In addition; presence of hypertension, OP and probable sarcopenia were independently related with SSP tears in postmenopausal women.

In the literature, the incidence of RC tears is reported to increase with aging [24, 25]. With older age, loss of muscle mass and strength (i.e. sarcopenia), degeneration of the tendons and strain over long periods can easily lead to RC tears [26]. The number of micro vessels in the RC are significantly lower in the elderly people which makes tendons more vulnerable to fibrovascular hyperplasia, fat formation, calcification and atrophy—eventually increasing the tear risk [27]. However, the specific causes and mechanism of RC tears/degeneration remain unclear. Our study revealed that age was the most significant factor related with SSP tears in postmenopausal women.

Rotator cuff (RC) tears have multifactorial etiology including anatomic/genetic factors, age-related degeneration, tendon hypovascularity and traumatic injuries [28]. With increasing age, RC becomes more susceptible to injury, degeneration and poor healing [24, 25, 27]. Similarly, our results also showed that the incidence of RC tears increased with age. On the other hand, degenerative tears are mainly present in the hypovascularized area called the distal critical zone close to the insertion of SSP [29]. Considering the fact that poor regeneration contributes to degeneration, all pathologies involving the micro vessels and causing hypoxia of the critical zone (e.g., obesity, hypertension, DM and hyperlipidemia) are likely to be associated with RC disease [1, 30]. Supportingly, in 408 patients who underwent arthroscopic RC tear repair, hypertension was found to be a significant risk factor for the occurrence (about twofold higher) and severity of the tear [28]. Likewise, our results have shown that hypertension (about 1.6 times higher) was an important predictor for SSP tears. Additionally, in a retrospective study including 148 patients who underwent arthroscopic RC repair, the obese group showed higher re-tear rates [31]. Another comparative study including 232 pre- and postmenopausal women showed that RC tears were associated with higher BMI [32]. Similarly, high values of BMI were positively related with SSP tears in our study.

Bone is composed of type I collagen (~ 40% of its volume) and minerals (~ 50% of its volume), and tendon contains type I collagen (90% of its volume), water and small amounts of proteoglycan [33]. Estrogen deficiency after menopause affects bone and tendon tissues alike/simultaneously [34]. Accordingly, BMD and tendon thicknesses decrease by age, especially after menopause [35]. Estrogen level has a direct effect on collagenous tissue, and its deficiency is related with the decrease in tensile strength [36], collagen synthesis, fiber diameter and, density-lowering proliferation of tenocytes and increased tendon degradation [37, 38]. In addition, estrogen deficiency induces loss of bone on the humeral head and affects the entheseal architecture. Therefore, estrogen deficiency can lead to OP and tendon thinning/tears in postmenopausal women. In our study, when we excluded bilateral SSP tears, we observed that SSP tendons were found to be thinner in women who had unilateral SSP tear. Although age-related tendon degeneration and tears also/frequently occurred in men, one study including men and women aged 45–89 years revealed that female gender is a risk factor for massive RC tears [39]. Additionally, a recent study investigating RC re-tear rates in men and women aged 33 to 79 years has shown that re-tear rates after RC repair were more common in women—possibly related to the regional BMD decline in women after menopause, [40, 41]. These data appear to be in line with our study results.

Alterations in (non)collagenous proteins play important role in age- and disease-related biomechanical changes in bone and tendon tissues [42]. It has been found that BMD has an effect on the tendon’s failure and strength [40, 43]. A cadaveric study has revealed that RC tear is directly related to the humeral head BMD [9]. Further, BMD of the humeral head affects RC repair healing whereby higher BMD is related with better healing [10, 11]. In this aspect, (pre)clinical studies have shown that anti-OP treatments can improve BMD at the RC enthesis and enhance the biomechanical properties of tendon repairs [40, 44, 45]. As tendons with higher proportions of large collagen fibrils have higher tensile strength [46]; together with the known association between fiber diameter and tendon stiffness [47], bone loss may be a sufficient risk factor for SSP thinning/tears. Therefore, improving proximal humeral BMD in OP may help to enhance the RC strength and to decrease the re-tears after repair [40]. Although a longitudinal study revealed that the risk of RC tears (evaluated by magnetic resonance imaging and US) was about 1.8-fold higher in OP patients than those without [12]; our results showed that OP increased the risk of SSP tears by about 1.4 times. When regression analysis was made as newly diagnosed OP and those receiving antiresorptive treatment, this rate increased to 1.7 times in patients under treatment compared to those without OP. In addition, the fact that OP was associated with SSP tears at a lower rate in our study might have been caused due to the exclusion of secondary OP causes (steroid use, rheumatic and neurological diseases etc.) and the evaluation of only SSP (but not the other RC tendon) tears.

It is well known that cortical bone is also affected by OP and some studies showed that it could be related to RC tears and proximal humerus fractures [48, 49]. Similarly, in a previous study, Achilles tendon ruptures with avulsion of the calcaneal bone was found to be associated with decreased calcaneal BMD [43]. Accordingly, we have investigated the relationship between Achilles tendon thickness and OP; however, we did not observe any significant result. This finding can be explained by the fact that ankle is a weight-bearing joint while shoulder is not. Hence, the tendons covering each joint can be affected differently by body weight and activity status. Likewise, a comparative study reported that proximal humerus BMD values were lower than femoral neck and the difference had a positive correlation with BMI [50]. It can be attributed to the fact that non-weight-bearing nature of the proximal humerus makes it more prone to regional OP. Therefore, lumbar and proximal femur BMD measurements can underestimate the BMD of proximal humerus, especially in overweight patients [51].

The grip strength is considered to be a measure of general health, frailty, fitness and exercise adequacy. Studies have found that low grip strength was independently associated with BMD in lumbar spine and hip [52, 53]. A study including 546 postmenopausal women reported that among sarcopenia-related parameters, having low grip strength (i.e., probable sarcopenia) was independently associated with the presence of OP [19]. We also found that probable sarcopenia was independently related with about 1.4 times higher SSP tears in postmenopausal women.

Previous studies have shown larger tendon cross-sectional areas after heavy (vs. light) resistance exercises but not after prolonged endurance training [54]. It is also reported that regular/intense exercise increases bone formation [55] and tendon size [54]. In our study, frequency of exercise was lower in women with SSP tears than those without, but the majority of our participants were sedentary. In other words, exercise-related data were based only on self-reports whereby frequency/intensity could be subjective (possibly inappropriate for comparison). Likewise, moderate-intensity physical activity in postmenopausal women with OP could have not been enough to increase the bone formation [55] or the tendon size [56].

There are a few limitations of our study. First, its design was cross-sectional and therefore temporality problems cannot be completely resolved. Further, two-thirds of our subjects were on drug-free follow-up or newly diagnosed OP patients. Therefore, disease duration/severity could have led to an underestimation as regards the predictive role of OP in tendon tears. Another limitation would be the lack of analyses concerning the impact of antihypertensive drugs and statins. Yet, our patients were using different combinations of them.

In conclusion, our study showed that age, presence of hypertension, probable sarcopenia and OP were independently related with SSP tears in postmenopausal women. Although OP appeared to be directly related to 1.4 times higher SSP tears, US examination for SSP tear/thickness can be recommended for OP patients, especially those who has any risk factors such as older age, hypertension and low grip strength. Indisputably, longitudinal studies in larger groups are definitely required to assess the impact of different medications (commonly used in these patients) on SSP tendon tears.
